# Physiology of deletion mutants in the anaerobic β-myrcene degradation pathway in *Castellaniella defragrans*

**DOI:** 10.1186/1471-2180-12-192

**Published:** 2012-09-04

**Authors:** Frauke Lüddeke, Aytac Dikfidan, Jens Harder

**Affiliations:** 1 Dep. of Microbiology, Max Planck Institute for Marine Microbiology, Celsiusstr. 1, 28359, Bremen, Germany

**Keywords:** β-myrcene, phellandrene, Anaerobic degradation, Geraniol dehydrogenase, Linalool dehydratase-isomerase, Genetic system

## Abstract

**Background:**

Monoterpenes present a large and versatile group of unsaturated hydrocarbons of plant origin with widespread use in the fragrance as well as food industry. The anaerobic β-myrcene degradation pathway in *Castellaniella defragrans* strain 65Phen differs from well known aerobic, monooxygenase-containing pathways. The initial enzyme linalool dehydratase-isomerase *ldi*/LDI catalyzes the hydration of β-myrcene to (*S*)-(+)-linalool and its isomerization to geraniol. A high-affinity geraniol dehydrogenase *geoA*/GeDH and a geranial dehydrogenase *geoB*/GaDH contribute to the formation of geranic acid.

A genetic system was for the first time applied for the betaproteobacterium to prove *in vivo* the relevance of the linalool dehydratase-isomerase and the geraniol dehydrogenase. In-frame deletion cassettes were introduced by conjugation and two homologous recombination events.

**Results:**

Polar effects were absent in the in-frame deletion mutants *C. defragrans* Δ*ldi* and *C. defragrans* Δ*geoA*. The physiological characterization of the strains demonstrated a requirement of the linalool dehydratase-isomerase for growth on acyclic monoterpenes, but not on cyclic monoterpenes. The deletion of *geoA* resulted in a phenotype with hampered growth rate on monoterpenes as sole carbon and energy source as well as reduced biomass yields. Enzyme assays revealed the presence of a second geraniol dehydrogenase. The deletion mutants were *in trans* complemented with the broad-host range expression vector pBBR1MCS-4*ldi* and pBBR1MCS-2*geoA*, restoring in both cases the wild type phenotype.

**Conclusions:**

In-frame deletion mutants of genes in the anaerobic β-myrcene degradation revealed novel insights in the *in vivo* function. The deletion of a high-affinity geraniol dehydrogenase hampered, but did not preclude growth on monoterpenes. A second geraniol dehydrogenase activity was present that contributes to the β-myrcene degradation pathway. Growth on cyclic monoterpenes independent of the initial enzyme LDI suggests the presence of a second enzyme system activating unsaturated hydrocarbons.

## Background

Monoterpenes represent a prominent group of volatile organic compounds (VOC), with an estimated mean global emission of 117 Tg C yr^-1^ into the atmosphere [1] and a fast photochemical turnover [2]. Especially coniferous plants are considered to be main producers of monoterpenes, e.g. for thermotolerance or for communication between plants or the interaction between plants and insects [3-5]. Monoterpenes also enter the soil by the rhizosphere or by rotten leafs [6], where they inhibit growth of microorganisms as well as of seedlings [7-9], but also stimulate the bacterial activity detectable in higher biomass and CO_2_-production [5,10,11]. By definition, monoterpenes possess a carbon skeleton based on two C_5_ units originating from isopentenyl pyrophosphate (IPP), which is synthesized via the mevalonate (in eukaryotes) or the mevalonate-independent pathway (in prokaryotes and plant plastids) [12-14]. Mainly, plant monoterpenes are produced via the latter pathway, but the metabolic cross linkage between both has been reported in several species [15,16].

Monoterpenes are together with sesquiterpenes the major constituents of essential oils. Due to their status – they are generally recognized as safe (GRAS) [17] - and their odorous properties, these substances are widespread in the food, cosmetics, flavour and fragrance industry [18]. Monoterpenes are utilized as energy and carbon source by several aerobic microorganisms, a fact known since the 1960s [19-21]. Most reports dealt with *Pseudomonas* species, e.g. [22-28], but also *Bacillus stearothermophilus*[29], *Rhodococcus erythropolis*[30], and *Enterobacter cowanii*[31] metabolize these hydrocarbons. The microbial degradation of α-pinene and limonene, one of the most widespread monoterpenes in nature, involve complex and multiple pathways that comprise in large part oxidation reactions [30,32-34]. In addition these studies revealed the importance of oxygenases, which catalyze hydroxylation reactions with molecular oxygen as co-substrate [35-38].

**Figure 1:**
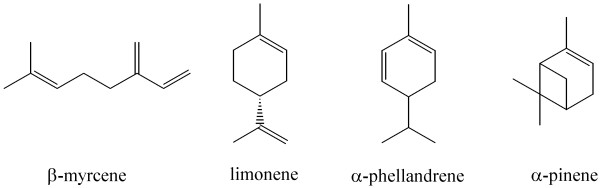
**Monoterpene substrate range of*C. defragrans ***[40].

**Figure 2:**
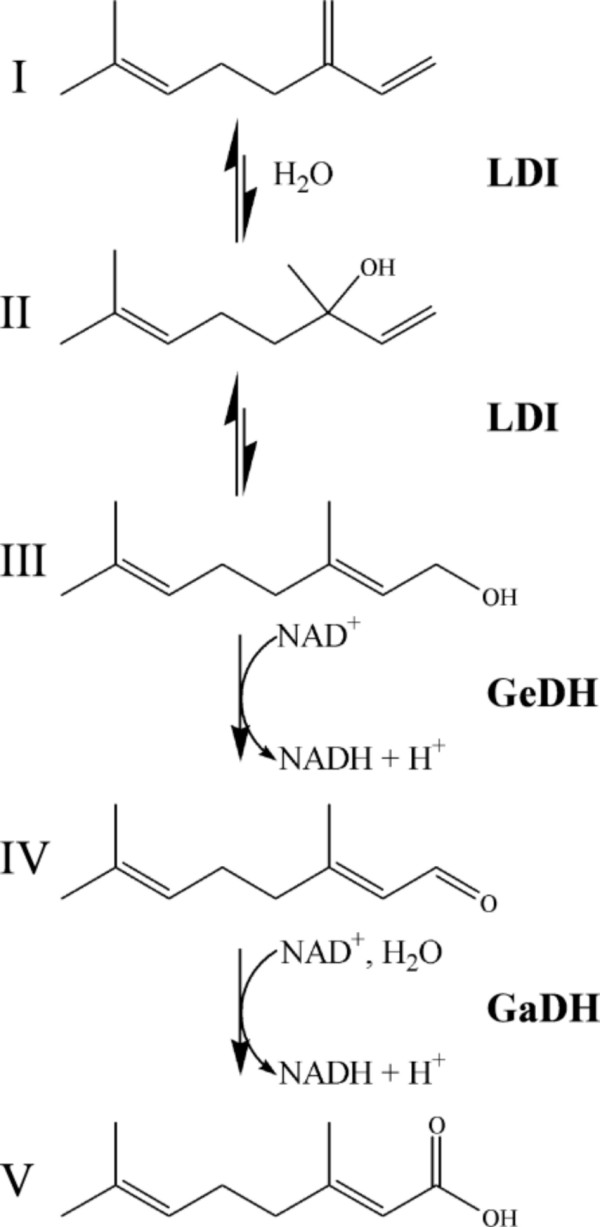
**Anaerobic degradation pathway of β-myrcene by*C. defragrans*.** Anaerobic β-myrcene degradation in *C. defragrans* 65Phen. I, β-myrcene (7-methyl-3-methylen-1,6-octadien); II, (S)-(+)-linalool; III, geraniol ((*2E*)-3,7-dimethyl-2,6-octadien-1-ol); IV, geranial ((2E)-3,7-dimethyl-2,6-octadien-1-al); V, geranic acid ((2E)-3,7-dimethyl-2,6-octadienoic acid). LDI, linalool dehydratase-isomerase; GeDH, geraniol dehydrogenase; GaDH, geranial dehydrogenase.

Under anaerobic conditions, the biochemistry for the activation of these natural abundant alkenes seems to follow a totally different mechanism. The first evidence for the anaerobic degradation of monoterpenes were seven nitrate-reducing enrichment cultures with monoterpenes as sole carbon source [39]. Isolation led to the description of four *Alcaligenes defragrans* strains, including strain 65Phen isolated with α-phellandrene [40]. A taxonomic study transferred these strains in the novel genus *Castellaniella* within the *Alcaligenaceae*, as *C. defragrans*[41]. The betaproteobacterium is capable of degrading a broad substrate range of a-, mono-, and bicyclic monoterpenes (Figure 1) [40]. Initial metabolite studies on the anaerobic monoterpene degradation pathway in *C. defragrans* elucidated the demand for a sp^2^-hybridized C1-atom as structural prerequisite for monoterpenes utilization [42] as well as the formation of geranic acid as intermediate [43], which is likely degraded on a modified β-oxidation pathway [44,45]. These findings proposed the degradation of β-myrcene via hydration to linalool, followed by isomerisation to geraniol, and then two oxidations to geranial and to geranic acid [43]. The genes and proteins involved this pathway were recently identified [46,47] (Figure 2). The bifunctional linalool dehydratase-isomerase *ldi*/LDI catalyzes the first two steps, the highly enantiospecific hydration of β-myrcene to (*S*)-(+)-linalool and its isomerisation to geraniol [46,48]. Subsequently, two dehydrogenases oxidize the allylalcohol geraniol and geranial. The geraniol dehydrogenase *geoA*/GeDH (E. C. 1.1.1.183) is a member of the medium-chain dehydrogenase/reductase superfamily [49] with high affinity for its substrate geraniol [47]. *In vitro* studies confirmed the activity of a geranial dehydrogenase *geoB*/GaDH. Both dehydrogenases were expressed in cells growing with monoterpenes [47]. 

So far, the evidence for the anaerobic β-myrcene degradation pathway was rather biochemically based on metabolite and enzyme studies. To prove the physiological role *in vivo*, we created deletion mutants of *C. defragrans* missing the gene *ldi* and *geoA*, respectively. The previous findings, i.e. the geranic acid formation and the induced dehydrogenase activities, were observed in both acyclic and monocyclic monoterpenes grown cells and suggested the existence of a common degradation pathway. To clarify whether there is one defined metabolic route or multiple pathways present for the anaerobic degradation of monoterpenes in *C. defragrans*, we deleted the initial, β-myrcene-activating enzyme, the LDI. The deletion of the GeDH was of interest due to the frequent presence of multiple alcohol dehydrogenases in genomes, often with a broad substrate range.

## Results and Discussion

### Construction of the in-frame deletion mutant *C. defragrans* Δ*ldi* and Δ*geoA*

Growth of *C. defragrans* as single colony under denitrifying conditions was achieved on acetate in a defined, solidified medium. A spontaneous mutant strain resistant to rifampicin (150 μg/ml) was obtained showing the phenotype of the wildtype with respect to growth on monoterpenes (Additional file 1: Table S1). Conjugation was established with the broad host range plasmid pBBR1MCS-2, proceeding with a frequency of 1.8 × 10^-4^ transconjugants cell/ donor cells in 8 h (Additional file 1: Table S2). The plasmid was maintained in *C. defragrans.* For genomic deletion mutants, we constructed pK19mobsacBΔ*ldi* and pK19mobsacBΔ*geoA* that carried the start and stop codon of the *ldi* (ORF26) or *geoA* (ORF31) separated by a specific restriction site and the upstream and downstream located regions (Additional file 1: Figure S1). The sequence information was obtained from a 50 kb contig (Acc. no. FR669447.2) with the following annotation for ORFs adjacent to *ldi* or *geoA*: ORF27 as a thioesterase, ORF29 as a putative subunit of cytochrome c oxidase, ORF30 as a secretory protein and ORF32 as a long-chain-fatty-acid CoA ligase, while for ORF25 only hypothetical proteins were found in database queries (Additional file 1: Figure S 2). Conjugation and homologous recombination yielded genomic in-frame deletions, with a second recombination frequency of 0.5% and 1.25% for the deletion of *ldi* and of *geoA*, respectively. Analysis by PCR revealed in the deletion mutants the expected, shortened amplicons with primer pairs spanning the deleted gene in comparison with the wild type (Additional file 1: Figure S3). Polar effects due to the deletion of *ldi* or *geoA* were not detected in mRNA analyses (Additional file 1: Figure S4). The genes *ldi* or *geoA* and their native ribosomal binding site were cloned in the MCS of pBBR1MCS plasmids. Conjugation into *C. defragrans* deletion mutants yielded ampicillin-resistant transconjugants named *C. defragrans* Δ*ldi*comp and kanamycin-resistant transconjugants named *C. defragrans* Δ*geoA*comp.

### Physiological characterization of *C. defragrans* Δ*ldi*

**Figure 3:**
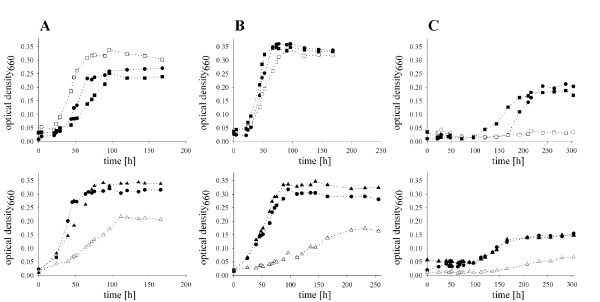
**Time courses of anaerobic denitrifying growth of *C ****.****defragrans *mutant strains.** Time courses of anaerobic, denitrifying growth of *C. defragrans* strains 65Phen (●), Δ*ldi* (□), Δ*ldi*comp (■), Δ*geoA* (▵) and Δ*geoA*comp (▴) on different carbon sources, namely (**A**) 4 mM α-phellandrene, (**B**) 4 mM limonene, and (**C**) 4 mM β-myrcene. Negative controls without inoculum or without substrate did not show an increase in turbidity (data not shown).

Under standard culturing conditions for anaerobic, denitrifying growth with 10 mM nitrate and 4 mM cyclic α-phellandrene or limonene in 2,2,4,6,6,8,8-heptamethylnonane (HMN), *C. defragrans* strains 65Phen, Δ*ldi*, and Δ*ldi*comp grew to final OD ranging from 0.25 to 0.35 (Figure 3A, B). *C. defragrans* strains 65Phen metabolized the acyclic β-myrcene, but *C. defragrans* Δ*ldi* lacking the gene for the *ldi* failed to grow with this substrate (Figure 3C). The *in trans* complementation Δ*ldi*comp restored the wild type phenotype. These data showed that the LDI is essential for the metabolism of β-myrcene, but not for the cyclic monoterpenes α-phellandrene and limonene.

In previous studies, β-myrcene as well as α-phellandrene supported the formation of geranic acid in cell suspension experiments. The geranic acid pool was 10fold larger in β-myrcene experiments than with the cyclic monoterpenes α-pinene, α-phellandrene, and limonene [43]. We assayed the geranic acid pools in *C. defragrans* mutant strains under nitrate-limited conditions in liquid cultures on 6 mM monoterpene in HMN (Table 1). This metabolite was only detectable in myrcene-grown *C. defragrans* cultures with the *ldi* either present in the genome or *in trans*, in concentrations of 8.85 μM and 6.61 μM, respectively. In α-phellandrene grown cultures, geranic acid was detectable in media of these *C. defragrans* strains in concentrations of 0.24 μM and 0.33 μM. Geranic acid formation was not detectable in cultures of the mutant lacking the gene *ldi*. The RP-HPLC detection limit was 6.4 nM, thus geranic acid formation in *C. defragrans* Δ*ldi* was below a thousandth of that in the wild type. Growth on α-phellandrene clearly does not involve the formation of geranic acid suggesting the presence of another monoterpene degrading pathway that circumvents the activation of the substrate by LDI as well as geranic acid formation. 

**Table 1:** Geranic acid pools in cultivation media

***C. defragrans strains***	**Geranic acid concentration [μM]**	
	**α-Phellandrene**	**β-Myrcene**
65Phen (wild type)	0.24 ± 0.01	8.85 ± 0.6
Δ*ldi*	n.d.	n.d.
Δ*ldi*comp	0.33 ± 0.24	6.61 ± 0.19
Δ*geoA*	n.d.	4.96 ± 1.58
Δ*geoA*comp	0.89 ± 0.25	11.79 ± 0.31

*C. defragrans* cultures were grown in 150 mL with 6 mM α-phellandrene or β-myrcene and 10 mM nitrate at 30°C and 130 rpm. Inoculum size was 1% (v/v). Duplicate determination. Detection limit for geranic acid was 6.4 nM. n.d. = not detectable.

Under aerobic conditions microbial biotransformation of (−)-limonene and β-myrcene revealed the formation of enantiopure (−)-perillyl alcohol, perillyl acid and myrcenic acid [30,50-52]. Anaerobic hydroxylations catalyzed by molybdenum enzymes have been recently reported, e.g. the hydroxylation of ethylbenzene to (*S*)-phenylethanol in *Aromatoleum aromaticum*[53] and of cholesterol to cholest-1,4-diene-3-one in *Sterolibacterium denitrificans*[54]. Whether the degradation of cyclic monoterpenes proceeds via a homologue pathway is subjected in ongoing research. To our knowledge, this is the first report on the existence of different activation mechanisms for cyclic and acyclic monoterpenes in one bacterial strain.

### Physiological and enzymatic characterization of *C. defragrans* Δ*geoA*

The deletion of *geoA* resulted in an increased generation time and reduced biomass yields, e.g. on α-phellandrene, limonene and β-myrcene (Figure 3A-C, Table 2). Nitrate was completely consumed, but the generation time was always prolonged, e.g. 3.5-fold for α-phellandrene. The biomass formed as determined by protein analyses was decreased by 32% to 48% in the deletion mutant (Table 2). Most likely, geraniol was oxidized at slower rate in the deletion mutant. This seems to have an inhibitory effect on the growth due to the known geraniol *in vivo* toxicity of above 5 μM in the aqueous phase [47]. The intracellular geraniol concentrations were below the detection threshold of gas chromatographical analysis, but we observed physiological evidence for increased geraniol pools. In the cultivation system with HMN, 4 mM geraniol stopped monoterpene utilization completely [47]. In the wild type, addition of 16 mM acetate supported growth in the presence of 4 mM geraniol and 20 mM nitrate to an OD_660_ of 0.15 (± 0.002; n = 2). The deletion mutant *C. defragans* Δ*geoA* also grew after acetate addition, but reached only an OD_660_ of 0.061 (± 0.01; n = 2), although both strains consumed the same nitrate amount. In conclusion, *C. defragans* Δ*geoA* reacts more sensitive towards geraniol than the wild type. 

**Table 2:** **Physiological properties of *C. defragrans *strains growing with different monoterpenes **

	**α-Phellandrene**			**Limonene**			**β-Myrcene**		
	**65Phen**	**Δ*geoA***	**Δ*geoA*comp**	**65Phen**	**Δ*geoA***	**Δ*geoA*comp**	**65Phen**	**Δ*geoA***	**Δ*geoA*comp**
MaxOD_660_	0.321	0.217	0.342	0.318	0.174	0.347	0.155	0.066	0.149
Generation time [h]	9.8	34.9	13.5	25.4	50.8	44.9	46.9	57.1	45.8
NO_3_^-^ consumed [mM]	10	10	10	10	10	10	7.3	5.8	8.1
NO_2_^-^ formed [mM]	0	0	0	0	0	0.01	0.22	0	0.009
Biomass formed [g/L]	0.34	0.23	0.32	0.35	0.22	0.35	0.14	0.08	0.17

*C. defragrans* strains 65Phen (wild type), Δ*geoA* and Δ*geoA*comp were grown under standard conditions at 28°C for 280 h (α-phellandrene, limonene) or for 304 h (β-myrcene) with 4 mM monoterpene (in HMN) and 10 mM nitrate. As negative control served a culture without inoculum.

The growth phenotype of the wild type was recovered in the mutant strain by complementation with the *geoA* gene located on a broad-host range plasmid. The *in trans* complemented mutant *C. defragrans* Δ*geoA*comp revealed physiological characteristics similar to *C. defragrans* 65Phen: growth rate and yield, monoterpene consumption and nitrate reduction were almost identical suggesting that the wild type phenotype was restored by GeDH constitutively expressed from the plasmid pBBR1MCS-2*geoA* (Table 2, Figure 3).

The absence of GeDH was expected to reduce the rate of geranic acid formation. In this study, geranic acid was detected in cultures grown on 6 mM monoterpene in the presence of HMN and 10 mM nitrate (Table 1). Cultures were sampled after nitrate depletion. Geranic acid concentrations of acidified and lysed cultures were 9 ± 1 μM in the medium of the wild type and 12 ± 1 μM in the medium of the complemented mutant, but only 5 ± 2 μM in the medium of *C. defragrans* Δ*geoA*, thus revealing a limited capacity to form geranic acid in the absence of GeDH.

**Figure 4:**
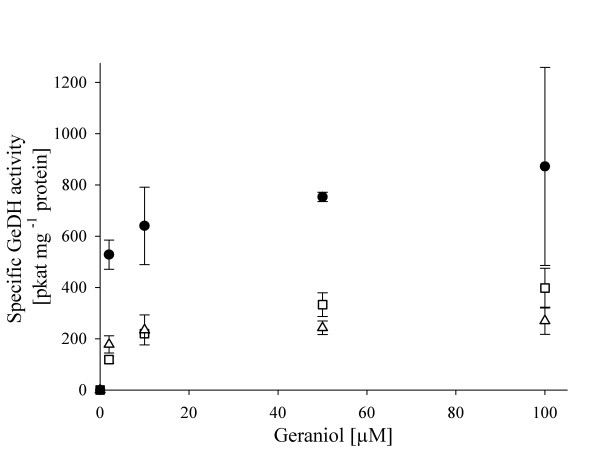
**Initial specific GeDH activity of *C. defragrans *strains 65Phen, Δ*geoA*and Δ*geoA*comp.** The initial specific GeDH activity was measured in triplicates with cytosolic, cell-free extracts of *C. defragrans* strains 65Phen (□), Δ*geoA* (Δ) and Δ*geoA*comp (●). Geraniol concentrations tested were 0, 2, 10, 50, 100 μM.

The *ΔgeoA* phenotype has still the capacity to degrade monoterpenes, an indication for the presence of another alcohol dehydrogenase that catalyzes the geraniol oxidation. Thus, we tested the GeDH activity spectrophotometrically in cell-free, cytosolic extracts of *C. defragrans* strains 65Phen, Δ*geoA* and Δ*geoA*comp. Under standard conditions, with 0.8 mM geraniol as substrate and identical protein concentrations in the assay, the geraniol oxidation rates were 5.8 nkat mg^-1^ protein for *C. defragrans* 65Phen and 1.05 nkat mg^-1^ protein for *C. defragrans* Δ*geoA*. Complementation restored the activity to 9.4 nkat mg^-1^ protein in *C. defragrans* Δ*geoA*comp. The *in vivo* concentration of geraniol inside the cell is expected to be in the micromolar range [47]. The GeDH activity in the extracts of *C. defragrans* Δ*geoA* catalyzed the reaction with a high affinity, the apparent concentration for half-maximal rate was below 10 μM geraniol (Figure 4). This indicated an activity of the second alcohol dehydrogenase at physiological conditions. 

In summary, the presented data argue for a reduced geraniol flux to geranic acid in the metabolism of the deletion mutant. We suggest that a geraniol accumulation or increased pools of metabolites derived from geraniol on other pathways cause a reduced growth rate as indicated by prolonged generation time, decreased biomass production, and reduced geranic acid formation. The accumulation of a toxic intermediate in monoterpene catabolism causing reduced growth rate has also been seen for deletion mutants of *P. putida* M1 in ß-myrcene degradation [24,55]. Accumulation of geraniol is known to be toxic for cells: due to its hydrophobic properties it can integrate into bacterial membranes causing disintegrations followed by failure of the proton motive force [56,57].

The presence of several ADHs in a genome is not unusual. In microorganisms, alcohol dehydrogenases possess a wide variety of substrate specificities and are involved in different physiological functions [58]. For various ADHs deficient mutants, retarded growth on the prevailing substrate and reduced ADH activity was observed [59-61]. Also in plants the existence of additional ADHs capable of oxidizing geraniol was suggested [62].

## Conclusions

We developed a genetic system for *Castellaniella defragrans* and constructed in-frame deletion mutants that allows for insights into the physiology of the anaerobic degradation of monoterpenes.

*C. defragrans* Δ*geoA* lacking the gene for a geraniol dehydrogenase was physiologically analysed. The *geoA* deficient strain exhibited reduced growth on monoterpenes and slower geraniol oxidation rates in soluble extracts, in comparison to the wild type. The original phenotype was restored *in trans* with an episomal *geoA* in the *C. defragrans* Δ*geoA*comp. One explanation for the reduced growth is a higher steady-state level of geraniol in the cell causing toxic effects. These observations together with reduced geranic acid formation demonstrate clearly a participation of GeDH in the anaerobic degradation of β-myrcene. However, the *geoA* deletion is not mortal. A second GeDH activity is present in soluble extracts. This suggests a need for both GeDHs to balance the geraniol formation by oxidation during fast growth of the wild type.

The physiological characterization regarding growth with acyclic and cyclic monoterpenes exhibited an unexpected effect of the *ldi* deletion that caused a phenotype dependent on the substrate structure in *C. defragrans* Δ*ldi*: the cyclic monoterpenes α-phellandrene and limonene were metabolized, but not the acyclic β-myrcene. Thus, the degradation of the acyclic β-myrcene required the activity of a linalool dehydratase-isomerase that was not necessary for the degradation of cyclic monoterpenes. This observation indicates for the presence of a second hydrocarbon activating system in *C. defragrans*.

## Methods

### Bacterial strains and plasmids

Table 3 described plasmids, *C. defragrans* strain 65Phen (wild type as well as derivatives) and *E. coli* strains used in this study. In course of the text, abbreviations are: i) *C. defragrans* 65Phen-RIF is equivalent to *C. defragrans* RIF; ii) *C. defragrans* 65Phen-RIF Δ*ldi* is equivalent to *C. defragrans* Δ*ldi*; iii) *C. defragrans* 65Phen-RIF Δ*ldi*comp is equivalent to *C. defragrans* Δ*ldi*comp; iv) *C. defragrans* 65Phen-RIF Δ*geoA* is equivalent to *C. defragrans* Δ*geoA*; v) *C. defragrans* 65Phen-RIF Δ*geoA*comp*geoA* is equivalent to *C. defragrans* Δ*geoA*comp.

**Table 3:** Strains and plasmids used in this study

**Strains or plasmids**	**Genotype, markers and further characteristics**	**Source/reference**
Strains		
* E. coli*		
S17-1	*Thi*, *pro*, *hsdR*, *recA* with RP4-2[Tc::Mu-Km::Tn7]	[63]
One Shot®Top10	F- *mcr*A Δ(*mrr-hsd*RMS*-mcr*BC) φ80*lac*ZΔM15 Δ*lac*X74 *rec*A1 *ara*D139 Δ(*araleu*) 7697 *gal*U *gal*K *rps*L (Str^R^) *end*A1 *nup*G	Invitrogen
* C. defragrans*		
65Phen	Wild type	[40]
65Phen-RIF^a^	Ra^R^	This study
65Phen-RIF Δ*ldi*^b^	Ra^R^, Δ*ldi*	This study
65Phen-RIF Δ*ldi*comp^c^	Ra^R^, Δ*ldi*, pBBR1MCS-4*ldi*	This study
65Phen-RIF Δ*geoA*^d^	Ra^R^, Δ*geoA*	This study
65Phen-RIF Δ*geoA*comp^e^	Ra^R^, Δ*geoA*, pBBR1MCS-2*geoA*	This study
Plasmids		
pCR4-TOPO	Am^R^, Km^R^, *lac*Zα	Invitrogen
pK19mobsacB	Km^R^, *sacB* modified from *B. subtilis*, *lac*Zα	[64]
pK19mobsacBΔ*ldi*	Km^R^, *sacB* modified from *B. subtilis*, *lac*Zα, ORF25, ORF27	This study
pK19mobsacBΔgeoA	Km^R^, *sacB* modified from *B. subtilis*, *lac*Zα, ORF29-30, ORF32	This study
pBBR1MCS-4	Am^R^ , *mob*, *lac*Zα	[65]
pBBR1MCS-4*ldi*	Am^R^, *mob*, *lac*Zα, *ldi*	This study
pBBR1MCS-2	Km^R^, *mob*, *lac*Zα	[65]
pBBR1MCS-2*geoA*	Km^R^, *mob*, *lac*Zα, *geoA*	This study

^a^ abbreviated in course of the text to *C. defragrans* RIF, ^b^ abbreviated to *C. defragrans* Δ*ldi*, ^c^ abbreviated to *C. defragrans* Δ*ldi*comp, ^d^ abbreviated to *C. defragrans* Δ*geoA*, ^e^ abbreviated to *C. defragrans* Δ*geoA*comp.

### Culturing conditions and growth media

*E. coli* strains were cultured according to established methods [66]. For propagation of plasmids, additional antibiotics were supplemented in the indicated concentrations [66]. Maintenance and growth experiments in liquid cultures with *C. defragrans* 65Phen and mutants were performed as described previously [40]. Growth in liquid cultures was monitored by turbidity measurements at 660 nm.

Minimal medium for plates contained 50 mM sodium acetate in medium solidified with 18 g/L agar and additionally buffered with 50 mM HEPES, pH 7.2. Incubation took place in anaerobic jars for 4 to 5 days under N_2_ atmosphere at 28°C. Biomass production of *C. defragrans* strains was performed according to [46].

Antibiotics were used at following concentrations (unless indicated otherwise): 50 μg/mL ampicillin, 50 μg/mL kanamycin, and 150 μg/mL rifampicin. Plating efficiency was determined by plating decading dilution-to-extinction series of cell suspensions with known optical density (OD) at 660 nm in duplicates.

### Preparation and manipulation of genetic material

Genomic DNA was isolated from *C. defragrans* 65Phen using the DNeasy Tissue Kit (Quiagen, Hilden, Germany). Plasmid DNA was isolated from *E. coli* strains and *C. defragrans* 65Phen using mini-plasmid preparation kits (Quiagen). Gel-excised PCR products and plasmid fragments were purified with the QIAquick gel extraction kit (Quiagen).

PCR amplification was usually performed using Taq polymerase (Promega, Madison, USA). For cloning purposes a mixture of Taq polymerase and a thermostable polymerase with proofreading activity (Fermentas, St. Leon Rot, Germany) were applied.

### Transcription analyses with Reverse Transcriptase-PCR

Preparation of total RNA from *C. defragrans* strains after growth on α-pellandrene was performed with RNeasy Mini Kit (Qiagen) according to manufacturer’s instructions, followed by cDNA synthesis using the Revert Aid™ First Strand cDNA Synthesis Kit (Fermentas). For transcriptional analyses, RT-PCR was performed with 35 cycles with primer pairs listed in Table 4. Negative controls included RT-PCR without reverse transcriptase. Table 4 lists primers used for the different amplification purposes.

**Table 4:** Oligonucleotide sequences used in this study

**Primer**	**Sequence (5`→ 3`)**	**Amplicon (bp)**	**Target gene**
*ldi* deletion construct			
(pK19mobsacB			
ORF25_*Eco*RI_F	TCGTAGAATTCCATGCCTGCGCACGCTGATG	1307	ORF25
ORF25_*Xho*I_ATG_R	GAGACTCGAGATGTTCAGTCGCATGTCGTCT		
ORF27_*Xho*I_TAA_F	TATACTCGAGTAAGGGGGACGCGGCGGCCTG	763	ORF27
ORF27_*Hind*III_R	TCGTAAAGCTTATGGACGACGGCACATGGA		
p27 + _F	ACGAAGCCGAGCATGCCCAC	2199	encompassing
p27 + _R	AGCAGCAGGCCGACGTGTTC		ORF27
p27mismatch_F	CGCCCGGTTCGAGGAAGG	-	nucleotide
p27mismatch_R	CCCTTCCTCGAACCGGGCG		exchange
*geoA* deletion construct			
(pK19mobsacB)			
ORF2930_*Xba*I_F	TCTAGACCACCAGGGCGCATGCTTCAGTT	1749	ORF2930
ORF2930_*Xho*I_R	CTCGAGTGAGCAGGGCGCGACTCC		
ORF32_*Xho*I_F	CTCGAGCATCGTTGAGTGTCTCCTGGTTG	1712	ORF32
ORF32_*Hind*III_R	AAGCTTTGGAAACGACATAGGGGACAGGA		
Control of *ldi* or *geoA* deletion			
* ldi*_F	CGCCACCACCGAGGACTATTTC	432	*ldi*
* ldi*_R	AGGTGGGCATGCTCGGCTTCGTA		
ORF25_401_F	GAAGGTGCGCGGCAAGGAATA	2463^a^/	ORF25-27
ORF27_2005_R	CATGGACAGCGGCACACGGGCAA	1269^b^	
* geoA*_260_F	ACCGGGTCGTGCTGTCCTTCAAT	284	*geoA*
* geoA*_527_R	CGCGCCGGTCTGGATGC		
ORF30_30967_F	CCAGACGCCGCCGATGATGAAGAG	1904^a^/	ORF30-32
ORF32_32822_R	TATCTGAACAAGCCCGAACTGACC	740^c^	
*ldi* complementation construct			
(pBBR1MCS-4)			
* ldi*_*Eco*RI_F	TGCGGAATTCATGCGGTTCACATTG	1206	*ldi*
* ldi*_*Bgl*II_R	CGCGAGATCTTTATTTCCCTGCGA		
*geoA* complementation construct			
(pBBR1MCS-2)			
* geoA*_*Xba*I_F	AATCTAGACGCCCTGCTCAGAACAC	1290	*geoA*
* geoA*_*Hind*III_R	GAGCAAGCTTACCCTGCGCAAGCAGTTC		
Control of adjacent gene transcription			
ORF25_254_F	CCCACCGGCTTCTCGTAGTC	535	ORF25
ORF25_788_R	GCAAGGGCCTGGGCGTGATGTC		
ORF27_28_F	CATGGACAGCGGCACACGGGCAA	350	ORF27
ORF27_377_R	CAGTGGACCTCGCCGTGGAAAT		
ORF30_315_F	CGCGGGCGGGATGGTGAT	411	ORF30
ORF30_725_R	CGGGCGGCGGGTTCGTT		
ORF32_608_F	CCTGGCGGCCGGACGACAT	462	ORF32
ORF32_1069_R	CGCCGGAAGGGGAAACGAC		

Restriction sites are underlined. Oligonucleotide primers derived from annotated 50 kb contig of *C. defragrans* 65Phen (Acc. no. FR669447.2) [47]. ^a^ wild type; ^b^ C. defragrans Δldi, ^c^ C. defragransΔgeoA.

### Ligation and transformation of plasmid constructs

Subcloning of PCR products into pCR4-TOPO® vector (Invitrogen, Darmstadt, Germany) was performed corresponding to manufacturer’s instructions. PCR products with inserted restriction sites and purified plasmids were digested with the appropriate restriction enzymes and separated by gel electrophoresis. Both digested plasmids and PCR products were gel excised and purified. For ligation reactions, an insert-vector ratio of 1:1, 3:1 or 10:1 was chosen. To this mixture, T4-ligase buffer (1x), ATP (25 μM) and T4-ligase (2.5 U) were added. Incubation was for 12–16 h at 12°C. Transformation of 5 or 10 μL of the ligation reaction to chemical competent *E. coli* strains S17-1 or Top10 was performed as described [67]. Single colonies growing on selective solid medium were picked and screened for the correct insert size by PCR applying M13 or T7 primers. Plasmids of positive tested clones were purified and served as sequencing templates.

### Construction of suicide plasmids

The 5`- and 3`-flanking regions of *ldi* or *geoA* and the start and stop codons of the deleted gene separated by an appropriate specific restriction site were inserted into the suicide vector pK19mobsacB [64]. Oligonucleotide sequences are listed in Table 4.

Initially, the flanking regions were amplified from genomic *C. defragrans* 65Phen DNA with primers adding restriction enzyme sites to the PCR-product. The 5`-flanking region to the *ldi* was obtained with the primer pair ORF25_*EcoR*I_F and ORF25_*Xho*IATG_R. During amplification of the 3`-flanking region with primer pairs ORF27_*Xho*I_TAA_F and ORF27_*Hind*III_R difficulties occurred due to a terminator structure in the genome sequence that was solved with a nested PCR approach. A 2.2 kb amplicon comprising ORF 27 was obtained with the primer pair p27plus_F and p27plus_R that served as template for the initial named primer with an increased initial denaturation time (from 4 min to 10 min). Sequencing of the 763 bp amplicon revealed a base exchange at position 373 from guanine to adenine causing an amino acid replacement from proline to threonine. This shift was revoked by a site directed mutagenesis approach using primer p27_mismatch_F and p27_mismatch_R in combination with ORF27_*Xho*I_TAA_F and ORF27_*Hind*III_R, respectively [68]. The particular amplicons were bond to each other in another reaction with the exterior primer pair. The 5`-flanking region of the *geoA* was obtained with the primer pair ORF2930_*Xba*I_F & ORF2930_*Xho*I_R and the *geoA* 3`-flanking region ORF32_*Xho*I_F & ORF32_*Hind*III_R.

The obtained products were subcloned into pCR4-TOPO (Invitrogen, Darmstadt, Germany) and yielded pCR4-ORF25, pCR4-ORF27, pCR4-ORF2930 and pCR4-ORF32. The sequence correctness of these constructs was confirmed by sequencing and restriction digests.

Subcloning vectors were double digested with the prevailing added recognitions site for restriction enzymes. The flanking regions were excised, purified and ligated via a three-piece-ligation into the suicide vector pK19mobsacB [64]. Sequencing of the obtained plasmids pK19mobsacBΔ*ldi* and pK19mobsacBΔ*geoA* was performed to ensure correct sequence of the flanking regions including the start and stop codons of the deleted genes.

### Construction of complementation plasmids

For construction of the *in trans* vector both, the *ldi* and the *geoA* was amplified from genomic DNA of *C. defragrans* 65Phen with primer pair encompassing the entired ORF, i.e. for the *ldi* primer pair *ldi*_*EcoR*I &*ldi*_*Bgl*II, and for *geoA geoA*_*Xba*I_F &*geoA*_*Hind*III_R (Table 4). Via the added restriction enzyme recognition sites the amplicon was inserted into the multiple cloning site of two different derivatives of the broad-host range vector pBBR1MCS [69]. For confirmation of correct gene insertion the obtained plasmids pBBR1MCS-4*ldi* and pBBR1MCS-2*geoA* was sequenced.

### Conjugational plasmid transfer

The donor strain, an overnight culture of *E. coli* S17-1 carrying the appropriate plasmid, and the recipient *C. defragrans* RIF were grown to late exponential phase and were mixed in several ratios (1:1, 1:5, 1:10) in a total volume of 20 μL and spread as a single drop on minimal agar. After incubation for 24 h at 28°C under oxic conditions the bacteria were resuspended in 1 mL liquid minimal medium. Dilution-to-extinction series were streaked out onto solid minimal medium supplemented with kanamycin and rifampicin and anaerobically incubated at 28°C for four days.

### Preparation of cell-free extracts and determination of enzyme activities

Soluble extract preparations of *C. defragrans* strains 65Phen, Δ*geoA* and Δ*geoA*comp were performed as described [46]. The geraniol dehydrogenase activity was monitored in a standard assay following the reduction of NAD^+^ to NADH at 340 nm as described [47]. Equal total protein amounts were applied as certified in a 200-μl aliquot by the method of Bradford [70] with BSA as standard protein; concentrations were corrected for the unusual high binding of the Coomassie stain to albumin [71].

### Chemical analyses of biomass, educts and products

Nitrate and nitrite was measured by HPLC as described by [72]. Based on the fact that protein accounts for 50% of the cell mass, the Bradford assay was applied in duplicates with two different dilutions to determine the total biomass yield [72]. Geranic acid formation was assayed in liquid cultures of *C. defragrans* strains after confirmed nitrate depletion (Merckoquant® test strips (Merck, Darmstadt, Germany)). 10 mL cell culture was acidified with H_3_PO_4_ (final concentration 0.1 M) and extracted with tert-butyl methyl ether in a 1:0.4 ratio (two biological replicates per strain). The ether extract was extracted with 0.1 M NaOH (1:1) and the aqueous phase was subjected twice to reverse-phase HPLC on a Nucleodur® C18 ISIS (4.6 mm × 250 mm, Macherey Nagel, Düren, Germany). Separation of the organic acid was performed with 1 mM H_3_PO_4_ in an isocratic water-acetonitrile eluent (45/55 (v/v)) at 1 mL/min and 25°C. Intermediary, the column was cleaned with water-acetonitrile (20/80 (v/v)). UV detection was performed at 215 nm.

## Competing interest

The authors declare that they have no competing interests.

## Authors' contributions

AD isolated the rifampicin resistant *C. defragrans* strains and assayed the conjugation frequencies. AD constructed pK19mobsacB*ΔgeoA* and obtained *C. defragrans* Δ*geoA*. FL obtained *C. defragrans ΔgeoA* and Δ*ldi* deletion mutants and constructed the pBBR1MCS-2 derivates. FL performed all the physiological experiments. FL and JH analysed the physiological experiments and wrote the manuscript. All authors read and approved the final manuscript.

## Supplementary Material

Additional file 1 Additional Material. Click here for file
