# Supplementary material for: Physiology of deletion mutants in the anaerobic β-myrcene degradation pathway in Castellaniella defragrans
Source: BMC Microbiol. 2012 Sep 4;12:192. doi: 10.1186/1471-2180-12-192 (PMC3490891; doi:10.1186/1471-2180-12-192)

BMC Microbiology 1536376629675241

Physiology of deletion mutants in the anaerobic β-myrcene degradation pathway in *Castellaniella defragrans*

- Additional Material -

Frauke Lüddeke

Email: flueddeke@web.de

Aytac Dikfidan

Email: aytac.dikfidan@mpimf-heidelberg.mpg.de

Jens Harder*

corresponding author, Email: jharder@mpi-bremen.de

Dep. of Microbiology, Max Planck Institute for Marine Microbiology, Celsiusstr. 1, 28359 Bremen, Germany

Additional Material

Determination of antibiotic sensitivity and isolation of spontaneous antibiotic-resistant strains of *C. defragrans* 65Phen.

Endogenous antibiotic resistance strains of *C. defragrans* 65Phen was determined by growth on solid medium containing ampicillin (50 µg/mL), chloramphenicol (20 µg/mL), gentamycin (15µg/mL), kanamycin (50 µg/mL), neomycin (100 µg/mL), rifampicin (150 µg/mL), spectinomycin (100 µg/mL), streptomycin (50 µg/mL), and tetracycline (20 µg/mL). Stock solutions were prepared according to standard instructions [58]. After liquid cultures containing chloramphenicol (5 µg/mL), neomycin (25 µg/mL), rifampicin (150 µg/mL), spectinomycin (15 µg/mL) or tetracycline (20 µg/mL) showed growth of *C. defragrans* 65Phen, they were further incubated at antibiotic's work concentration and isolated on solid minimal medium. Dilution-to-extinction series of the wild type strain were used to inoculate solid minimal medium plates in presence or absence of the appropriate antibiotic under anoxic conditions for 4-5 days.

C. defragrans RIF2 was propagated without selection pressure in antibiotic-free medium for five passages with inocula of 10% (v/v). Afterwards plating on solid medium with and without rifampicin revealed that the resistance was preserved in 78% of the bacterial cells forming colonies.

Additional Table 1 - Growth of *C. defragrans* strains 65Phen and RIF1 - RIF4.

Optical density increase of cultures of *C. defragrans* strain 65Phen (in the absence of rifampicin) and four rifampicin-resistant mutants *C. defragrans* RIF 1-4 (in the presence of rifampicin) grown with 4 mM monoterpene and 10 mM nitrate. The inoculum size was 10% (v/v). Incubation took place for 28 d.

	Maximum OD_{660} *C. defragrans* strains				
Substrates	65Phen	RIF 1	RIF 2	RIF 3	RIF 4
β-Myrcene	0.35	0.48	0.48	0.39	0.42
(+)-Limonene	0.68	0.55	0.61	0.65	0.57
(+)-α-Terpineol	0.22	0.19	0.14	0.16	0.21
(+)-2-Carene	0.42	0.24	0.28	0.30	0.28
(+)-3-Carene	0.27	0.24	0.34	0.28	0.27
(-)-α-Pinene	0.38	0.38	0.30	0.37	0.38
(+)-Sabinene	0.40	0.40	0.46	0.43	0.41

Determination of the transconjugation frequency with E. coli S17-1 pBBR1-MCS2

The donor strain, an overnight culture of *E. coli* S17-1 carrying pBBR1-MCS2, and the recipient *C. defragrans* RIF were grown to late exponential phase and were mixed in several ratios (1:1, 1:5, 1:10) in a total volume of 20 µL and spread as a single drop on minimal agar. After incubation for 3 h, 8 h or 24 h at 28 °C under oxic conditions the bacteria were resuspended in 1 mL liquid minimal medium. Dilution-to-extinction series were streaked out onto solid minimal medium supplemented with kanamycin and rifampicin and anaerobically incubated at 28 °C for four days. To determine the transconjugation frequency, donor and recipient strain were plated onto solid medium with and without antibiotics. Growth of *E. coli* S17-1 containing pBBR1MCS-2 was inhibited in the presence of rifampicin as well as growth of *C. defragrans* RIF in the presence of kanamycin. But transconjugants *C. defragrans* RIF carrying pBBR1MCS-2 grew in the presence of rifampicin and kanamycin. The appearance of pBBR1MCS-2 in transconjugants was confirmed by PCR as well as plasmid DNA isolation and analysis of restriction digests. Optimal conjugation conditions were observed at donor-to-recipient ratios of at least one-to-one and a mating time of 8 h (Additional Table 2).

Additional Table 2 - Conjugation frequencies (FC).

Conjugation frequency (F_C) of plasmid transfer of pBBR1MCS-2 from *E. coli* S17-1 into *C. defragrans* RIF. Frequencies are given as tranconjugants cells /donor cells for experiments with 5.6 x 10^8 donor cells. The standard variation was always below 15 %.

	3 h mating		8 h mating	
Recipient: donor ratio	Transconjugants [CFU/mL]	F_C	Transconjugants [CFU/mL]	F_C
1:1	3.22 x 10^3	5.76 x 10^{-6}	6.36 x 10^4	1.14 x 10^{-4}
1:5	1.83 x 10^3	3.26 x 10^{-6}	9.92 x 10^4	1.77 x 10^{-4}
1:10	3.24 x 10^3	5.78 x 10^{-6}	9.65 x 10^4	1.72 x 10^{-4}

Assessment of plasmid stability under non-selective conditions.

The stable maintenance of plasmid pBBR1MCS-2 in *C. defragrans* 65Phen was assayed by anaerobic growth of transconjugants in denitrifying liquid medium with α-phellandrene as substrate without any selection pressure. The cultures were transferred five times in non-selective media (for 48 generations) and the colony-forming units (CFU)/mL were determined by plating appropriate dilution series in the presence or absence of kanamycin revealing a resistance in over 99% of the colonies. This demonstrated the stable maintenance of pBBR1MCS-2 in *C. defragrans* RIF.

Additional Figure 1 - Physical and genetic map of the mobilizable suicide vector pK19mobsacBΔ*geoA*.

Physical and genetic map of the mobilizable suicide vector pK19mobsacBΔ*geoA*. The narrow-host range vector lacks the capability of autonomous replication in *Betaproteobacteria* [58] and features selection marker genes encoding for kanamycin resistance (Km^R) and for sucrose sensitivity (*sacB*) are indicated in black. Genes encoding for the 5` and 3`flanking regions of the *geoA* are indicated in grey (ORF29, ORF30, ORF32). OriT = origin of transfer replication. Restrictions sites used for cloning are marked.

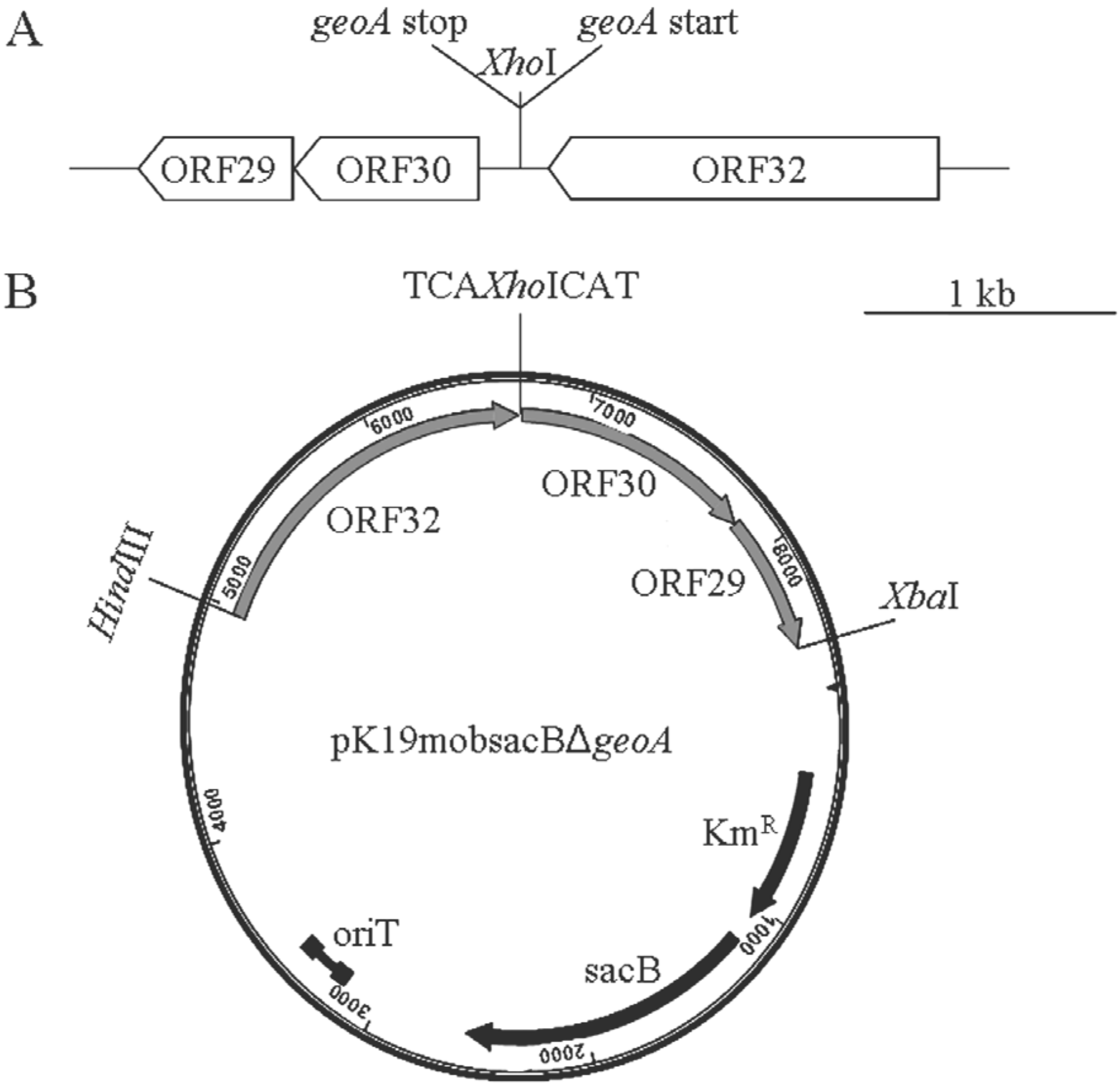

Additional Figure 2 - Genetic map of contig FR6694457.2

An annotation of open reading frames, including ORF 18 (*geoB*, geranial dehydrogenase), ORF 26 (*ldi*, linalool dehydratase isomerase) and ORF 31 (*geoA*, geraniol dehydrogenase). P indicates potential promoter regions, S Shine-Dalgarno sequences and T termination structures.

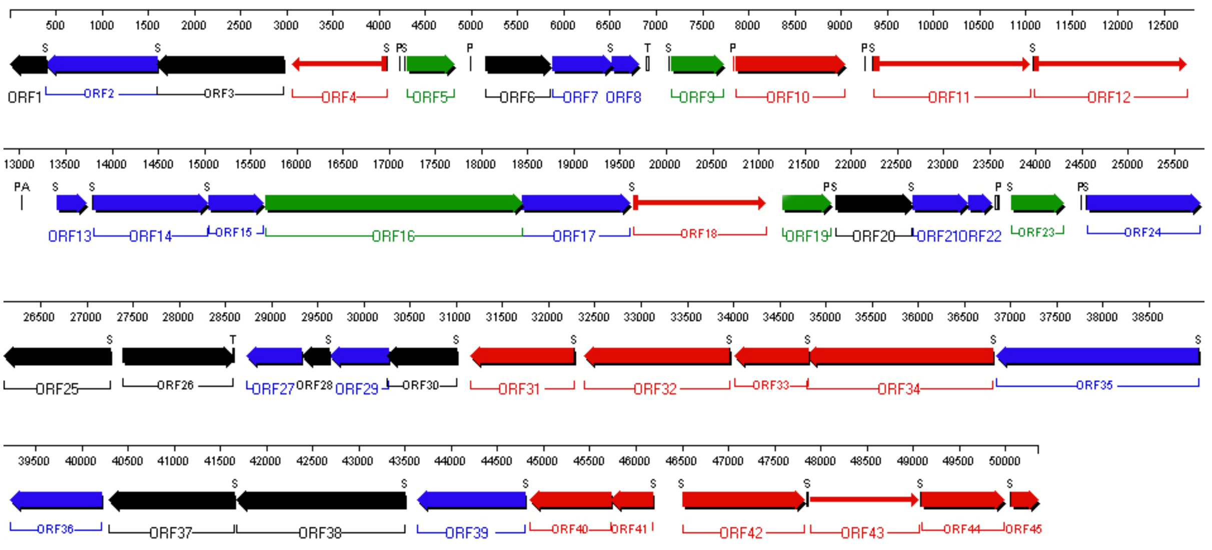

Additional Figure 3 - Genetic characterization of *C. defragrans* Δ*ldi* and *C. defragrans* Δ*geoA*.

Electropherogram of PCR products obtained with genomic DNA from *C. defragrans* strains 65Phen, Δ*ldi*, Δ*ldi*comp, Δ*geoA* and Δ*geoA*comp. The *ldi* deletion was confirmed with the primer pair ORF25_401_F & ORF27_2005_R that yielded in the case of the wild type a 2463 bp amplicon, and in the case of the *ldi*-deletion a 1269bp amplicon. The *geoA* deletion was detected with the primer pair ORF30_30967_F & ORF32_32822_R; the wild type yielded a 1904 bp amplicon, while the amplicon of *C. defragrans* Δ*geoA* is shortened to 740 bp. M = GeneRuler™ 100 bp Plus DNA Ladder (Fermentas).

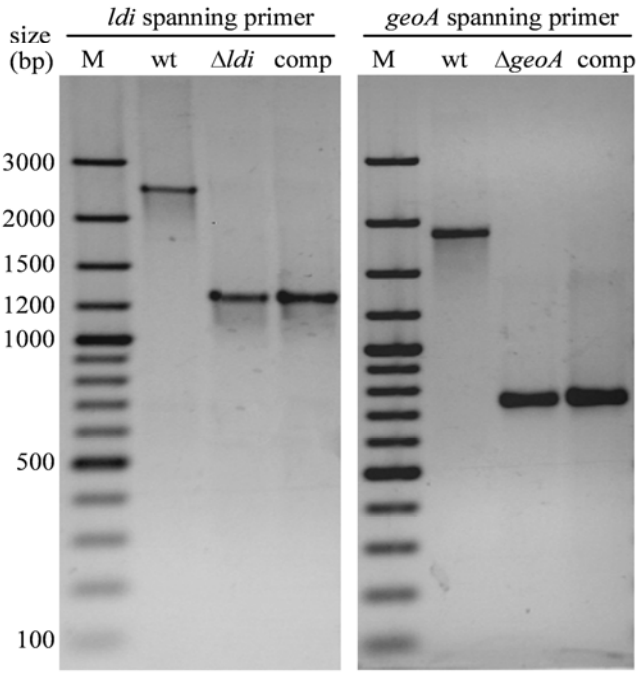

Additional Figure 4 - Transcriptional characterization of *C. defragrans* ∆*ldi* and *C. defragrans* ∆*geoA*.

As template for transcriptional analyses served cDNA obtained with gene-specific primers. RT-PCR was performed with primers annealing on A) *ldi*, B) ORF25 and ORF27, C) *geoA*, and D) ORF30 and ORF32. The transcriptional analysis revealed presence of the genes in the wild type and the complemented mutant, but their absence in the prevailing deletion mutant *C. defragrans* Δ*ldi* or *C. defragrans* Δ*geoA* (A, C). The expression of 5`- and 3`-flanking regions of the *ldi* (B).or *geoA* (D) was similar to that of the wild type. All negative controls, including a RT-PCR without reverse transcriptase, did not show the formation of amplicons (data not shown). M = GeneRuler™ 100 bp Plus DNA Ladder (Fermentas).

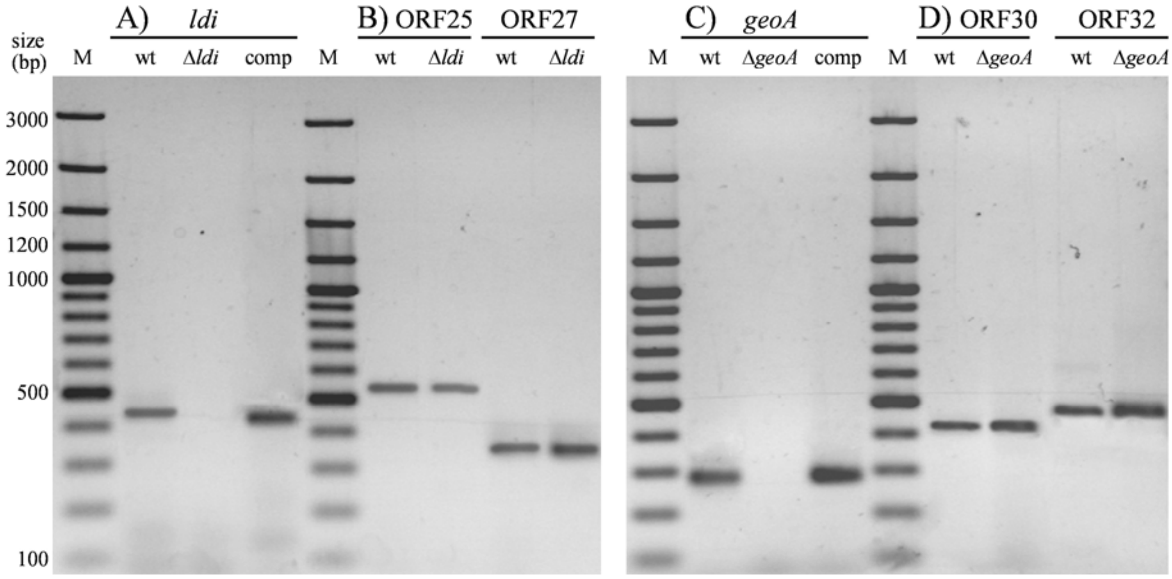

Supplement: Additional file 1 — Additional Material. [file 1471-2180-12-192-S1.pdf]
